# Design of High-Specificity Nanocarriers by Exploiting Non-Equilibrium Effects in Cancer Cell Targeting

**DOI:** 10.1371/journal.pone.0065623

**Published:** 2013-06-26

**Authors:** Konstantinos Tsekouras, Igor Goncharenko, Michael E. Colvin, Kerwyn Casey Huang, Ajay Gopinathan

**Affiliations:** 1 Department of Physics, University of California Merced, Merced, California, United States of America; 2 Department of Chemistry and Biochemistry, University of California Merced, Merced, California, United States of America; 3 Department of Bioengineering, Stanford University, Stanford, California, United States of America; Brandeis University, United States of America

## Abstract

Although targeting of cancer cells using drug-delivering nanocarriers holds promise for improving therapeutic agent specificity, the strategy of maximizing ligand affinity for receptors overexpressed on cancer cells is suboptimal. To determine design principles that maximize nanocarrier specificity for cancer cells, we studied a generalized kinetics-based theoretical model of nanocarriers with one or more ligands that specifically bind these overexpressed receptors. We show that kinetics inherent to the system play an important role in determining specificity and can in fact be exploited to attain orders of magnitude improvement in specificity. In contrast to the current trend of therapeutic design, we show that these specificity increases can generally be achieved by a combination of low rates of endocytosis and nanocarriers with multiple low-affinity ligands. These results are broadly robust across endocytosis mechanisms and drug-delivery protocols, suggesting the need for a paradigm shift in receptor-targeted drug-delivery design.

## Introduction

Collateral damage to healthy tissues caused by therapeutic agent action remains a major problem for cancer therapy, limiting treatment efficacy and applicability, and ultimately compromising patient survival. Specificity can be enhanced via therapeutic agent-containing nanocarriers equipped with ligands that bind receptors selectively overexpressed in cancer cells [1]–[3]. Although this approach holds promise, with a number of drugs on the market [4] and ongoing research focusing on nanocarrier-ligand conjugates and on identifying viable receptors [5]–[8], the occurrence of side effects in nanocarrier-treated patients remains a limiting factor. The rational design of multivalent nanocarriers for optimal specificity therefore presents an intriguing and important challenge.

Several recent theoretical and experimental studies [9]–[15] have been motivated by the relevance of multivalent ligand-receptor binding for both nanocarrier design and biological processes such as immune system function. In the context of high-specificity cancer cell targeting, it has been shown that multivalent nanocarriers equipped with low-affinity ligands can achieve much higher coverage on cells with modestly higher receptor densities under equilibrium conditions [3], [16]. However, it is the number of nanocarriers internalized that is of therapeutic consequence and not merely the number in contact with the cell surface under idealized conditions of equilibrium. We expect then that specificity will be governed by the different time scales associated with endocytosis, ligand-receptor binding/unbinding, and the method of drug delivery. Even when one considers simple linear response near equilibrium, it has been found that the magnitude of the endocytosis rate sets the maximal specificity [3], pointing to the critical importance of kinetics. In fact, we find that both the magnitude of the endocytosis rate and its functional dependence on the number of bound ligands, which is governed by the molecular mechanism of endocytosis, have substantial effects on the specificity.

Furthermore, equilibrium-based studies cannot address different drug-delivery protocols, as these create time-dependent nanocarrier availabilities and thus preclude the existence of a steady state. In any event, a steady state is virtually impossible to achieve *in vivo*, where natural biological cycles, homeostasis mechanisms, and the practicalities of drug-administration regulations all conspire against it. We explore these issues with an approach examining the *kinetics* of nanocarrier-ligand complexes binding to healthy and cancer cells so as to identify drug design strategies that maximize targeting specificity. We show that there exist peaks in the specificity as a function of both affinity and number of ligands, implying the existence of optimal nanocarrier designs with a large number of very weakly binding individual ligands with ligand-receptor dissociation constants in the mM range. We further demonstrate that specificity can be increased by orders of magnitude by exploiting the kinetics of the system via targeting receptors that are slowly endocytosed only in groups and/or by a judicious tuning of the time dependence of drug delivery. Remarkably, the optimal design region of parameter space shows little variation even among significantly different drug-delivery protocols and endocytosis mechanisms, pointing to the existence of robust design principles.

## Methods

**Figure 1:**
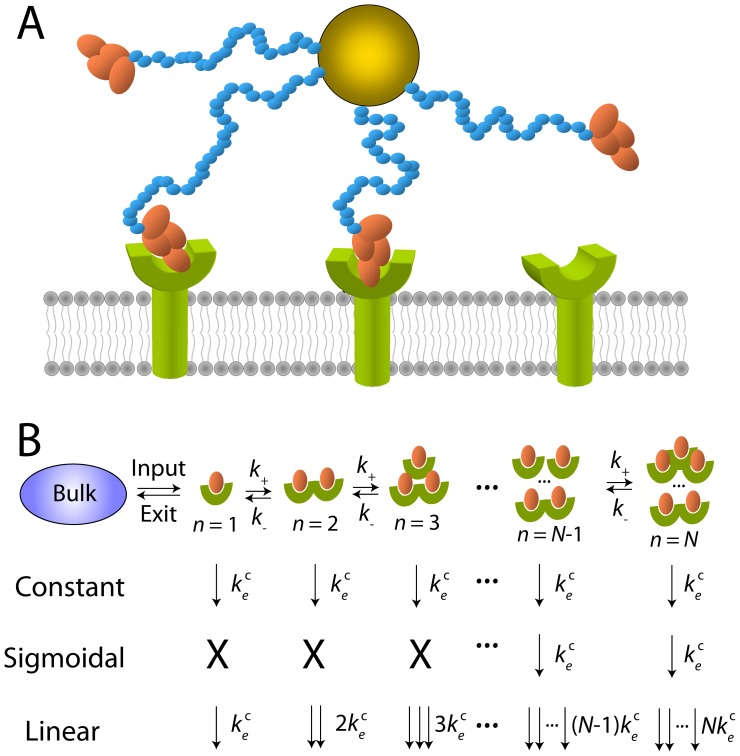
Schematic of model kinetics and endocytosis profiles. (A) Nanocarrier (gold) with two of four ligands bound on receptors (green). (B) Mapping to a 1D random walk with constant, sigmoidal, and linear profiles dictating rates of endocytosis in each ligand binding state 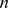.

We construct a minimal kinetic model for the endocytosis of nanocarriers equipped with *N* ligands (Fig. 1). Ligands independently bind to and unbind from individual receptors with rates 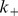, 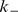 for healthy cells and 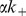, 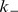 for cancer cells, where 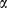 is the factor by which cancer cells overexpress receptors. The values of these rates depend on a variety of factors such as nanocarrier size and ligand/receptor type, and can be related to standard chemical kinetics constants by noting that 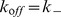 and 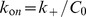, where 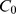 is the effective receptor concentration in the vicinity of a single ligand (File S1) and it is assumed that there is a large excess of mobile receptors. Therefore 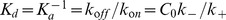, where 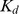 is the dissociation constant and 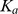 is the ligand-receptor affinity constant. The independence of the ligand binding, while assumed for simplicity, is a good approximation for many multivalent supramolecular interactions [17]. Nanocarriers enter the system with an input rate, 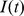, determined by the *drug-delivery protocol*, which is the method whereby the treatment is administered and the kinetics of first binding of the nanocarriers to the cell surface. We examined three cases: (i) the steady state arising from a constant supply of nanocarriers, (ii) the result of a single, high-concentration dose, and (iii) pretargeted delivery in which a ligand complex is introduced first (at 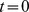) and then followed later by the active therapeutic (at 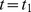), which docks with nanocarriers remaining in the system. For this last protocol, we measured specificity for a given *collection time window* 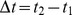, where 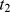 is set by physiological time scales and corresponds to the time beyond which endocytosis of activated therapeutic-nanocarrier complexes is negligible. After attaching to a cell, nanocarriers are removed from the system if all ligands unbind (we do not consider nanocarrier “recycling”) or if they are endocytosed. Endocytosis occurs with a rate 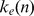, whose dependence on the number 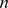 of bound ligands we term the *endocytosis profile*.

Each nanocarrier can then be represented by a 1D random walker on the discrete interval 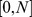 between an absorbing barrier at 0 and a reflecting barrier at *N*, with a rate 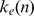 of particle loss at each site 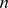 (Fig. 1B). At time 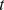, a walker at position *n* represents a nanocarrier with *n* ligands bound. Denoting the probability that a nanocarrier has *n* ligands bound at time *t* as 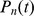, we derived master equations for a nanocarrier attached to a healthy cell:

for 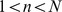,
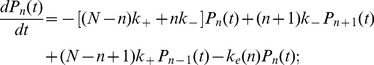
(1)for 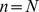,
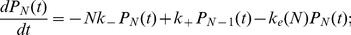
(2)for 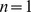,
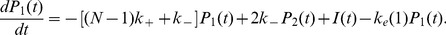
(3)For a nanocarrier attached to a cancer cell, 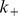 in Eqs. 1–3 is simply replaced by 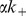.

This system of master equations can be recast as the vector equation
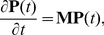
(4)where the 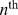 component of 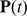 is 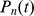 and the elements of the matrix 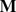 are dictated by the appropriate coefficients in the master equations. Eq. 4 can then be solved numerically, with initial conditions 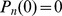, to determine the eigenvectors and eigenvalues for various combinations of endocytosis profiles, drug-delivery protocols, and values of 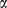, 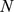, 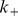, and (for pretargeted drug delivery) collection time windows 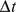. For each combination of drug-delivery protocol and endocytosis profile we determined the specificity, 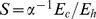, where 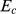 and 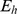 are the numbers of nanocarriers endocytosed by cancer and healthy cells, respectively. 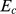 and 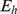 can be computed as the integral over the relevant time window of the net rate of drug internalization, 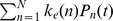. The factor 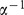 in the equation for 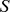 normalizes for the increased number of receptors on the cancer cell. In addition to determining the full numerical solutions for all combinations, we validated all results with Monte Carlo simulations using the Gillespie algorithm. Furthermore, we derived an analytical solution for the combination of a constant endocytosis profile at steady state in the limit 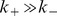 that agrees with our numerical results (File S1).

## Results

### Specificity peaks for high numbers of weakly bound ligands

**Figure 2:**
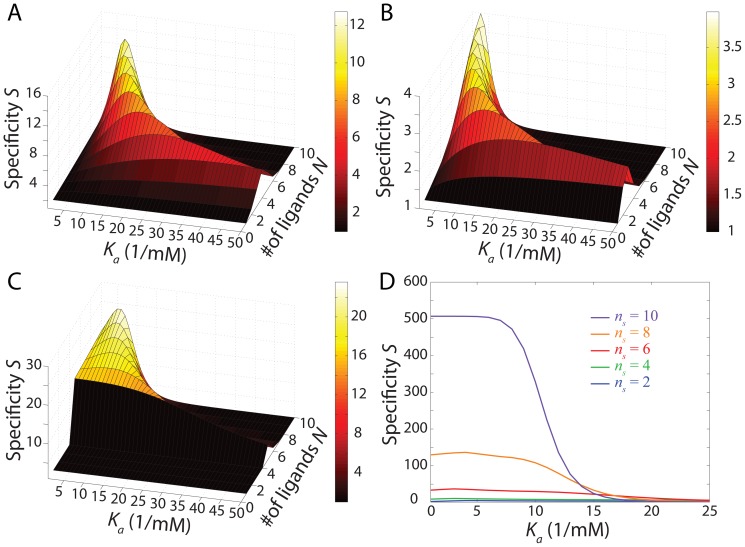
The endocytosis profile is a major determinant of specificity behavior. (A–C) Surface maps of specificity under the steady-state drug-delivery protocol with constant (A), linear (B), and sigmoidal endocytosis with 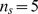 (C). (D) Maximum specificity under steady-state drug delivery and sigmoidal endocytosis for various threshold values. In all cases, the total number of ligands is 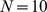, 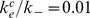, and 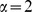.

To investigate the role of kinetic effects on the specificity, we first examined the impact of varying endocytosis profiles for a fixed steady-state drug-delivery protocol. We are interested in how the specificity 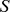 depends on the following subset of nanocarrier design parameters: the ligand number 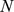 and the ligand-receptor affinity constant 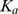. Fig. 2A displays the specificity for a constant endocytosis profile, where 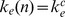. There is a well defined range of optimal design parameters where the specificity is substantially higher than the surroundings. Locally optimal solutions appear in a continuous curve ranging from small peaks at 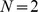 for large 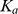 (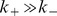) to large peaks at large 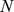 for 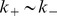.

In general, for reasonable values of 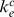 and 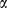 (Table 1), we find that specificity peaks globally for high numbers of ligands and a dissociation rate 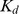 on the order of mM (Fig. 2A). This can be understood in terms of the relative values of the single ligand on and off rates. If 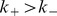, nanocarriers will bind to both healthy and cancer cells, while if 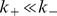 nanocarriers will bind to neither cell type; both effects reduce specificity. If 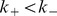, nanocarriers on healthy cells remain more likely to unbind than to bind a ligand; however, if 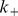 is close to 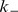 such that 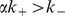, then nanocarriers on cancer cells will be more likely to bind than to unbind a ligand. Thus, in this regime, ligand binding selects for nanocarriers bound to cancer cells, and the desired bias increases as this pre-selected population feeds into the next ligand-binding reaction. Therefore, on average nanocarriers spend more time bound to cancer cells compared with healthy cells and thus have a higher likelihood of being endocytosed. For very large 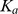, nanocarriers strongly prefer to bind rather than unbind a ligand irrespective of whether they are bound to healthy or cancer cells, giving a very high probability that a nanocarrier is nonspecifically endocytosed. This probability increases with the number of ligands, leading to the specificity decreasing and tending to 1 (the lower bound) in the large 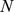 limit. The specificity at 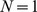 is identically unity, so as 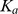 increases, the maximum tends to approach 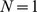, with the discreteness of 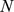 setting the optimum at 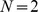 as 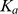 goes to infinity, a result that is confirmed by our analytics (File S1).

**Table 1:** Typical parameter values.

Parameter	Symbol	Investigated values	Reported values
Overexpression factor	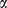	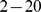	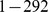 [22]
Endocytosis rate 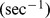	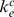	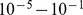	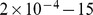 [3]
Dissociation rate 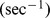	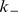	0.1	0.1 [23]
Logarithmic dissociation constant*	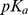	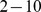	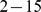 [24] **

*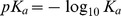, as measured for individual ligands.

** Multivalent complexes have been engineered with individual ligand 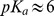, and full complex 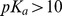. [25]

We next considered the effects of altering the functional form of the endocytosis profile. Figs. 2B and C show the specificities for the linear endocytosis profile
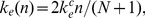
(5)and the sigmoidal endocytosis profile
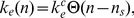
(6)where 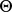 is the Heaviside step function and 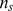 is the threshold value of 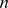, respectively. The location of the global optimum is not significantly affected by the changes to the endocytosis profile. At steady state, specificity values are similar for constant and linear endocytosis, the latter of which models the scenario where each ligand-receptor binding event triggers an independent signaling cascade that has a finite probability of resulting in endocytosis. By contrast, for sigmoidal endocytosis, the magnitude of the optimal specificity is considerably enhanced. For this cooperative endocytosis requiring multiple binding events [18], specificity is so enhanced because endocytosis occurs only with nanocarriers in ligand-binding states whose relative abundances display the cumulative effects of selection at every step. For a fixed total number of ligands, Fig. 2D demonstrates that specificity sharply increases by orders of magnitude as the value of the threshold, 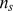, required to trigger endocytosis increases. This effect is a direct consequence of the specificity depending on the kinetics of binding, unbinding, and endocytosis. These results indicate that targeting receptors that are endocytosed in a cooperative fashion can yield significant improvements in specificity.

**Figure 3:**
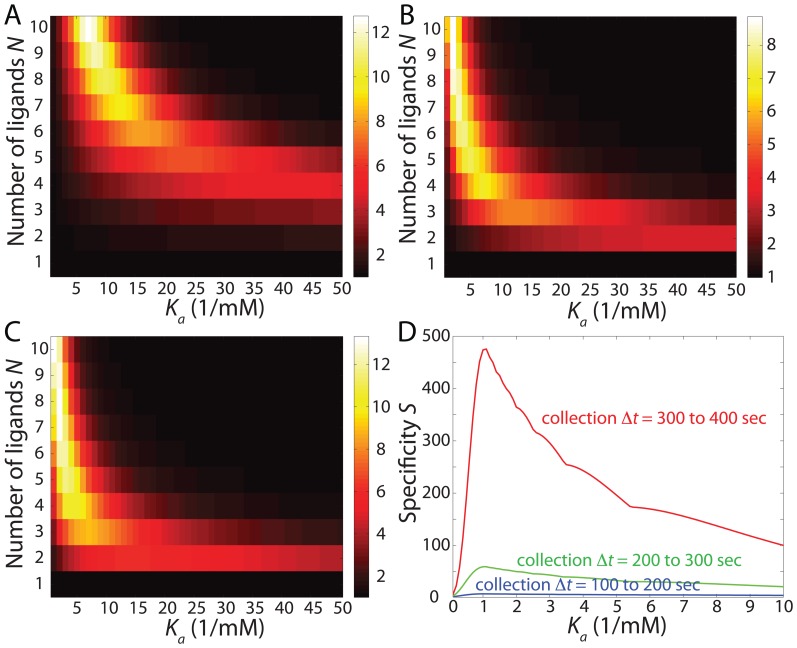
The drug-delivery protocol is also a major determinant of specificity behavior. (A–C) Specificity for a constant endocytosis profile under steady-state (A), single-dose (B), and pretargeted drug-delivery protocols (C). For the pretargeted drug-delivery protocol, the collection time window 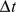 is from 200 to 300 seconds. (D) Maximum specificity under the pretargeted drug-delivery protocol and constant endocytosis for various values of 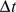. In all cases 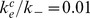 and 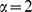.

We next examined the impact of varying drug-delivery protocols for a fixed constant endocytosis profile, again focusing on how the specificity 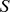 depends on the nanocarrier design parameters. Figs. 3A, B, and C show the specificity as a function of 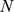 and 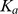 for the steady state, single-dose, and pretargeted drug-delivery protocols, respectively. Remarkably, just as in Fig. 2, we observed a robust set of locally optimal solutions appearing in a continuous curve ranging from 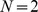 for 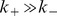 to large 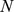 for 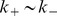. The fact that this optimal regime appears to be conserved across different protocols suggests that generically optimal drug designs, independent of administration protocol, are possible. For the pretargeted drug-delivery protocol for the same conditions, one can also achieve very large specificities simply through an appropriate choice of collection time window 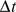: changes in 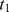 by a factor of 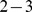 can result in orders of magnitude improvement in specificity (Fig. 3D). It should also be noted that specificity is much more sensitive to 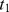 and only weakly dependent on 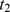.

### Multiplicative enhancement in specificity with optimal combination of drug-delivery protocol and endocytosis profile

**Figure 4:**
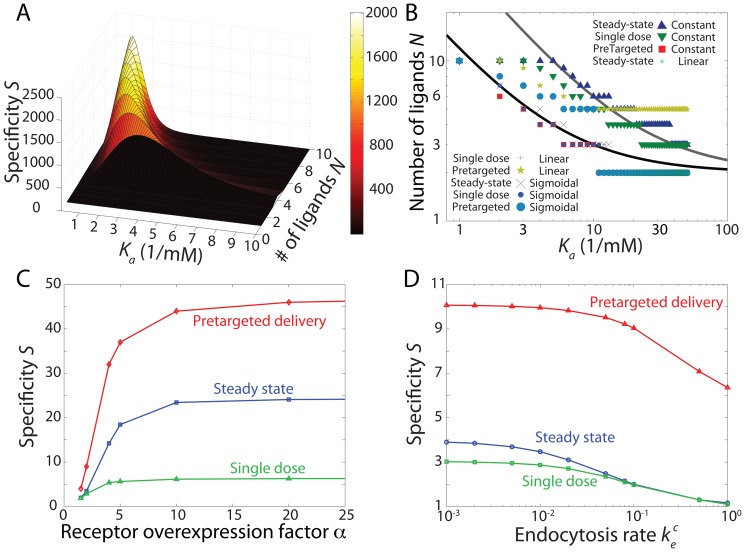
Specificity can be widely varied depending on parameter selection. (A) The combination of pretargeted drug-delivery protocol and sigmoidal endocytosis exhibits the highest specificity of all combinations examined. 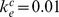, 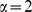, threshold at 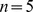. (B) Maximum specificity across all combinations. 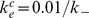, 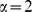, 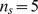 for sigmoidal endocytosis, and 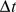 from 200 to 300 sec for pretargeted drug-delivery protocol. Solid lines correspond to 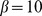 and 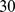. (C) Effects of the overexpression factor 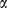 on specificity across drug-delivery protocols for constant endocytosis profile at 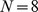, 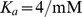. The specificity rises sharply at 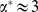. (D) Effects of the endocytosis rate 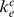 (normalized by 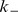) across drug-delivery protocols for constant endocytosis profile at 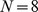, 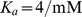, 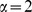.

Our results indicate that specificity can be significantly enhanced by independently tuning the drug-delivery protocol and the endocytosis profile via the choice of target receptor type. This suggests that judicious combinations of drug-delivery protocol and endocytosis profile coupled with appropriate design parameters could yield further increases in specificity. In Fig. 4A, we demonstrate this enhancement in the case of pretargeted drug delivery and sigmoidal endocytosis, for which we found a maximum specificity up to 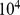 times the value of the selectivity expected from the overexpression factor alone and over 300 times the specificity for the combination of steady-state delivery and constant endocytosis, which is the closest mimic to equilibrium conditions. The effect appears to be multiplicative, with only modest (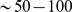) increases expected due to cooperative endocytosis (Fig. 2D) or pretargeted delivery (Fig. 3D) alone.

While the optimal region was broadly consistent across protocols or endocytosis profiles when one or the other was fixed, we wished to ascertain whether this remained true for arbitrary combinations. Fig. 4B depicts the maximum specificities for all combinations of drug-delivery protocol and endocytosis profile. There is a clear clustering of the optimal solutions for all combinations along a region ranging from 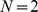 for large 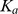 to large 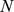 for low 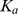. While we explained the 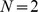 optimum at large 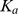, the global optimum for low 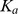 is of great interest. As discussed above, this regime should roughly correspond to the situation when a nanocarrier bound with one ligand to a cancer cell is marginally more likely to bind another ligand. This means 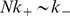 or 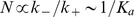. With the asymptote at large 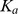 being 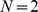, one arrives at the heuristic rule, 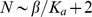, where 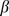 is a constant that depends on 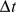, 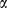, and 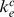. Fig. 4B shows two such curves with different values of 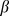 for comparison.

Since the optimal design solutions and the corresponding specificities depend on 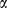 and 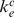, we show those dependencies in Figs. 4C and D across drug-delivery protocols for a fixed constant endocytosis profile. The specificity rises sharply for a value of 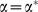 that is roughly constant across protocols. Since this value of 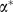 is set by the design parameters and choice of receptor (see File S1 for details), another strategy for achieving high specificity is to choose a receptor for which its typical overexpression factor is just above this value. This ensures that any cancer cells with 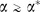 are targeted with high specificity. In fact, this was also identified as a mechanism for super-selectivity in equilibrium studies [16]. The kinetic effects of the drug-delivery protocol significantly affect the actual selectivity near 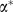, with the pretargeted protocol having the largest specificity enhancement. Finally, we observed that the specificity generally decreases with increasing endocytosis rate (Fig. 4D), indicating that receptors that are being recycled more slowly are potentially better targets. This particular dependence might also arise in a very different context: the accelerated establishment of morphogen gradients in the presence of degradation, which can be described by similar equations [19].

## Discussion

In conclusion, we have shown that specificity peaks occur as a function of both affinity and number of ligands, implying the existence of optimal nanocarrier designs. Overall, complexes with individual ligand 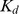 in the mM range (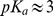) and large numbers of ligands per carrier have design features that yield high specificity in our simulations. This optimal region of the design parameter space is robust across drug-delivery protocols and endocytosis mechanisms. The precise specificity peak magnitude and sharpness can be customized through the careful selection of parameters or administration strategies. Possible strategies to increase specificity include targeting receptors that trigger endocytosis only if bound in groups and/or have a low overall endocytosis rate, and utilizing pretargeted drug delivery with long waiting times.

It should be noted that increased specificity comes at the cost of having a lower overall number of endocytosed nanocarriers. While we assumed that the dosage can be increased as necessary, in clinical practice there may be other factors such as the kidney's ability to clear the medication from the body that may limit the ability to maximize specificity. In order to address general design principles and strategies, we also chose to ignore a number of other specific system characteristics, including the possibility that 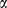 is time dependent, interactions between ligands, and details of the nanocarrier complex geometry (see File S1 for details).

Overall, our work complements equilibrium studies such as [16], by demonstrating that it is possible to design very high specificity complexes for non-equilibrium conditions. A specific advantage of our approach of using low-affinity multivalent nanocarriers is that it reduces the need for high-specificity ligands, which are usually of high molecular weight (such as antibody fragments) and can lead to drugs that have poor bioavailability [20]. It is to be noted that our work is not focused on a single biological system but rather addresses an entire class of biological, chemical, and bioengineering problems. By focusing on only physical quantities and not the specific chemical, biological, and clinical features of each possible system, we have derived broad, general, and overarching design principles. Our results can therefore potentially be applied to processes that trade off a few highly specific recognition sites versus a greater number of less specific sites; for example, to design a column to optimally separate chemicals or to design DNA sequences with an overrepresented number of certain motifs. It is also of interest to note that successive ligand-receptor binding reactions set up a multiple reaction step process similar to kinetic proofreading [21] that sharply increases the relatively low bias toward binding on cancer cells. Thus, as the number of steps increases, specificity can become very high. However, unlike in kinetic proofreading, energy is not expended to bias every reaction step, with the resulting cost being the overall reduction of nanocarrier endocytosis probability. Finally, given the ubiquity of multivalent low-affinity interactions and the inherently non-equilibrium environment in biology, it is possible that nature already exploits the mechanism we have discussed to gain significant improvements in specificity over the bounds dictated by equilibrium.

## Supporting Information

File S1Contains supporting information on assumptions in our model regarding receptor availability and ligand interdependence, rate dependencies, definitions of 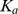 and 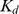, and an analytic solution of the master equations and determination of the specificity for the case of steady state with constant endocytosis profile. Figure S1, Surface plot of specificity 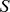 as a function of 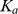 and 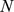, showing a ridge extending toward large values of 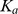 (out of the page). For fixed and large 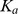, the specificity as a function of 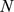 has a peak at 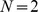. Here, 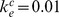 and 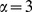. Figure S2, Crossover value of overexpression factor 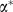 as a function of affinity, 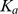, and number of ligands, 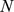 (inset). 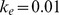 for both plots, 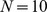 for the main plot and 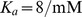 for the inset. In general, 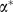 tends to qualitatively follow the behavior of the specificity 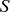 for the same parameter values.(PDF)
