# Supplementary material for: Design of High-Specificity Nanocarriers by Exploiting Non-Equilibrium Effects in Cancer Cell Targeting
Source: PLoS One. 2013 Jun 26;8(6):e65623. doi: 10.1371/journal.pone.0065623 (PMC3694107; doi:10.1371/journal.pone.0065623)
Supplement: File S1 — Contains supporting information on assumptions in our model regarding receptor availability and ligand interdependence, rate dependencies, definitions of and , and an analytic solution of the master equations and determination of the specificity for the case of steady state with constant endocytosis profile. Figure S1, Surface plot of specificity as a function of and , showing a ridge extending toward large values of (out of the page). For fixed and large , the specificity as a function of has a peak at . Here, and . Figure S2, Crossover value of overexpression factor as a function of affinity, , and number of ligands, (inset). for both plots, for the main plot and for the inset. In general, tends to qualitatively follow the behavior of the specificity for the same parameter values. (PDF) [file pone.0065623.s001.pdf]

Supporting Information for "Design of high-specificity nanocarriers by exploiting non-equilibrium effects in cancer cell targeting"

Konstantinos Tsekouras[1], Igor Goncharenko[2], Michael E. Colvin[3], Kerwyn Casey Huang[4*], Ajay Gopinathan[1*]

[1] **Department of Physics, University of California, Merced CA 95344, USA,**

[2] **New Economic School, Nakhimovskii Prospekt 47, Moscow 117418, Russian Federation,**

[3] **Department of Chemistry and Biochemistry, University of California, Merced CA 95344, USA,**

[4] **Department of Bioengineering, Stanford University, Stanford CA 94305, USA**

[*] **E-mail: agopinathan@ucmerced.edu; kchuang@stanford.edu.**

Assumptions regarding receptor availability and ligand interdependence

In this work, we assume for simplicity a sufficiently high receptor density and a nanocarrier-ligand complex of such a size that receptor availability is not a constraint. We also do not model exclusion effects, assuming that ligands are independent. The independence of the ligand binding, while assumed for simplicity, is nevertheless a good model for ligands bound to cargo by long tethers and is also a good approximation for many multivalent supramolecular interactions [1]. Our method can also be applied to particularly non-independent ligand cases by introducing effective functions $k_+(n)$, $k_-(n)$ that are functions of the number of ligands bound, via either a theoretical approach or the fitting of experimental data. We anticipate that the effect of this interdependence for cooperative binding would be similar to having a more sigmoidal endocytosis rate and hence a concomitant increase in specificity.

Rate dependencies

The values of the rates k_e^c, k_+, and k_- depend on a large variety of factors. k_e^c, the endocytosis rate, depends primarily on the type of receptor, but is also affected by factors such as nanocarrier size, geometry, chemical composition, and ligand type. k_+ and k_- depend primarily on the type of receptor targeted and the ligand, although nanocarrier size and geometry may affect these rates as well. In general, since our goal is to extract general principles, we treat these rates as free parameters and do not examine their dependencies or attempt to calculate them for a specific nanocarrier. To apply our approach to a specific nanocarrier, these rates would have to be determined by a theoretical calculation or inferred from experimental measurements.

Definitions of K_a and K_d

The ligand-receptor affinity constant is $K_a = K_d^{-1}$. Here, K_d is the dissociation constant for the ligand-receptor pair, given by

$$K_d = \frac{k_{\text{off}}}{k_{\text{on}}}.$$

where k_{off}, k_{on} are the conventional ligand-receptor off and on rates. We define $k_- = k_{\text{off}}$ and the *effective* on rate for a single ligand $k_+ = k_{\text{on}} C_0$, where C_0 is is the effective receptor concentration in the vicinity of a single ligand. This leads to

$$K_d = C_0 \frac{k_-}{k_+}.$$

We define r as the radius of the sphere a ligand can inscribe, which we assume is comparable to the size of a dendrimer (about 6 nm), and ρ as the density of receptors on a cell, taken to be 10^6 on a cell with diameter $10\,\mu\text{m}$. Assuming the cell is spherical, this yields a surface area of $10^{-10}\pi$ m^2. This yields an estimate for C_0 of

$$C_0 = \rho \frac{\pi r^2}{(1/2)(4/3)\pi r^3} = \frac{3\rho}{2r} \approx 1.3\,\text{mM}.$$

Analytic solution of master equations for steady state with constant endocytosis profile

If we assume that a steady state exists, then net particle sources and sinks in the system must balance. This yields

$$I = \sum_{n=1}^{N} k_e P_n + k_- P_1.$$

Applying this to the master equations at steady state, we obtain:

for $1 < n < N$,

$$0 = -[(N-n)k_+ + nk_-]P_n + (n+1)k_- P_{n+1} + (N-n+1)k_+ P_{n-1} - k_e P_n; \tag{1}$$

for $n = N$,

$$0 = -Nk_- P_N + k_+ P_{N-1} - k_e P_N; \tag{2}$$

for $n = 1$,

$$0 = -(N-1)k_+P_1 + 2k_-P_2 + k_e \sum_{n=2}^{N} P_n. \tag{3}$$

Using $\sum_{n=1}^{N} P_n = 1 \Rightarrow \sum_{n=2}^{N} P_n = 1 - P_1$ in Eq. 3,

$$0 = -[(N-1)k_+ + k_e]P_1 + 2k_-P_2 + k_e. \tag{4}$$

We also obtain $I = k_e + k_-P_1$. Eqs. 2 and 4 can then be solved to give

$$P_N = \frac{k_+P_{N-1}}{Nk_- + k_e} \quad \text{and} \quad P_1 = \frac{2k_-P_2 + k_e}{(N-1)k_+ + k_e}. \tag{5}$$

For Eq. 1, the discriminant is

$$\Delta = k_e^2 + 2nk_-k_e + 2(N-n)k_+k_e + [(N-n)k_+ - nk_-]^2 - 4(N+1)k_+k_-,$$

which can be simplified in the limit $k_+ \gg k_-$ to

$$\Delta \approx \Delta_s = [(N-n)k_+ + k_e]^2.$$

In general, it is difficult to determine whether Δ is positive, and a separate numerical evaluation should be made for each combination of N, k_+, k_-, and k_e^c. However, for $k_+ \gg k_-$ we can safely assume that the discriminant is positive and approximate it as Δ_s. In this regime, Eq. 1 has two roots

$$\rho_{1,2} = \frac{(N-n)k_+ + nk_- + k_e \pm [(N-n)k_+ + k_e]}{2(n+1)k_-},$$

which simplify to

$$\rho_1 = \frac{n}{2(n+1)} \quad \text{and} \quad \rho_2 = \frac{(N-n)k_+ + k_e}{(n+1)k_-} + \frac{n}{2(n+1)}. \tag{6}$$

For $2 \leq n \leq N-1$, solutions will be of the form

$$P_n = A\rho_1^{n-2} + B\rho_2^{n-2}$$

where A and B are constants. We have only one constraint, namely the normalization condition $\sum_{n=1}^{N} P_n = 1$.

As a result, we can arbitrarily set one constant. Choosing $B = 0$ yields results that do not agree with our Monte Carlo simulations, so we elect to set $A = 0$. For $N > 2$, this yields

$$P_N = B\frac{k_+}{Nk_- + k_e}\left(\frac{N-1}{2N} + \frac{k_+ + k_e}{Nk_-}\right)^{N-3} \quad \text{and} \quad P_1 = \frac{2Bk_- + k_e}{(N-1)(k_+ + k_e)} \tag{7}$$

with

$$B = \frac{(N-1)(k_+ + k_e) - k_e}{(N-1)(k_+ + k_e)}\left[B_1 + B_2 + B_3\right]^{-1}, \tag{8}$$

where

$$B_1 = \frac{2k_-}{(N-1)(k_+ + k_e)},$$

$$B_2 = \sum_{n=2}^{N-1}\left[\frac{n}{2(n+1)} + \frac{(N-n)k_+ + k_e}{(n+1)k_-}\right]^{n-2},$$

$$B_3 = \frac{k_+}{Nk_- + k_e}\left(\frac{N-1}{2N} + \frac{k_+ + k_e}{Nk_-}\right)^{N-3}.$$

For $N = 2$, since $P_1 + P_2 = 1$ it is simple to solve Eqs. 2 and 3 to obtain

$$P_1 = \frac{2k_- + k_e}{2k_- + k_e + k_+} \quad \text{and} \quad P_2 = \frac{k_+}{2k_- + k_e + k_+}. \tag{9}$$

We note that a similar analytical technique has been applied to a slight variant of this problem that was inspired by multi-state molecular motors [2].

Analytic determination of specificity for steady state with constant endocytosis profile

We are interested in calculating the specificity of nanocarriers for cancer cells, defined as the ratio of the number of nanocarriers internalized by cancer cells to the number of nanocarriers internalized by healthy cells per unit time. We refer to these two quantities as E_c and E_h respectively, and define X_c and X_h as the number of nanocarriers that have at least one ligand attached to a cancer or healthy cell, respectively. In general, X_c and X_h are functions of time, but at steady state they are constants. With α defined as the factor by which receptors are

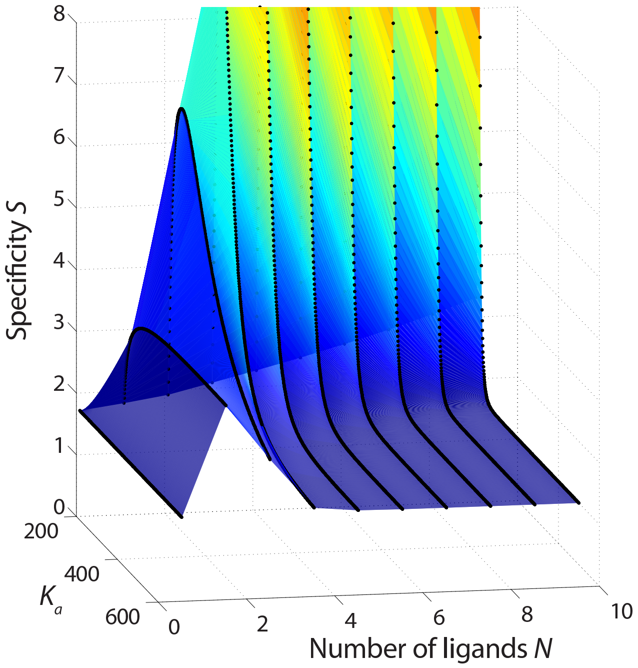

Figure S1 in File S1. Surface plot of specificity S as a function of K_a and N, showing a ridge extending toward large values of K_a (out of the page). For fixed and large K_a, the specificity as a function of N has a peak at $N = 2$. Here, $k_e^c = 0.01$ and $\alpha = 3$.

overexpressed on cancer cells, the specificity S is given by

$$S = \frac{1}{\alpha}\frac{E_c}{E_h} = \frac{1}{\alpha}\frac{X_c}{X_h}, \tag{10}$$

since at steady state $E_c = k_e X_c$ and $E_h = k_e X_h$. If both cancer and healthy cells are exposed to the same external concentration of nanocarriers C, at steady state the number of nanocarriers binding to a cell per unit time should balance the sum of nanocarriers internalized plus nanocarriers completely unbinding from the cell. Thus,

$$\alpha C = k_e X_c + k_- P_1^c X_c \tag{11}$$

and

$$C = k_e X_h + k_- P_1^h X_h, \tag{12}$$

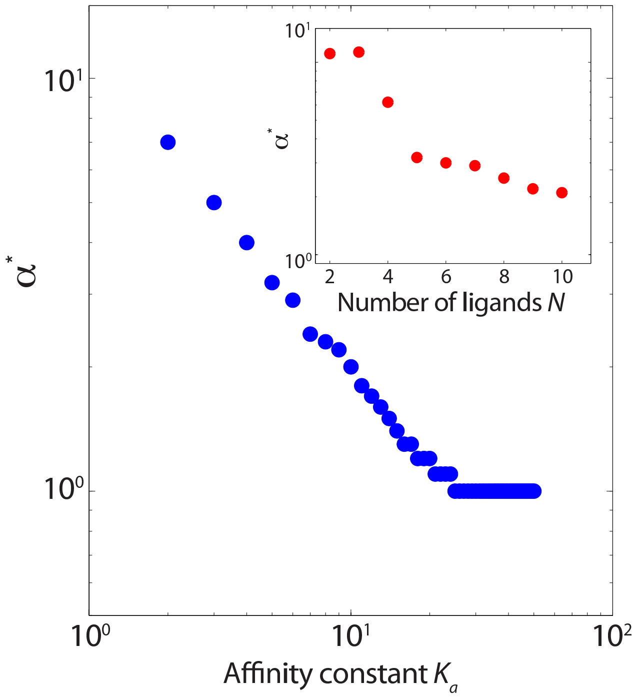

Figure S2 in File S1. Crossover value of overexpression factor α^* as a function of affinity, K_a, and number of ligands, N (inset). $k_e = 0.01$ for both plots, $N = 10$ for the main plot and $K_a = 8/\mathrm{mM}$ for the inset. In general α^* tends to qualitatively follow the behavior of the specificity S for the same parameter values.

where P_1^c, P_1^h are the probabilities that a nanocarrier will have only one ligand bound to a cancer or healthy cell, respectively. Solving Eqs. 11 and 12 for X_c and X_h and substituting into Eq. 10, we arrive at

$$S = \frac{k_e + k_- P_1^h}{k_e + k_- P_1^c}. \tag{13}$$

Combining Eqs. 7 and 13 yields an analytic result for the specificity at large K_a. Fig. S1 in File S1 shows the analytic solution, illustrating the peak at $N = 2$ for large K_a.

Dependence of α^* on K_a and N

We define α^* as the value of the overexpression factor α at which the specificity reaches 50% of its maximum value (taken to be the value of S at large $\alpha = 25$). Fig. S2 in File S1 shows how α^* depends on the design parameters N and K_a at steady state with constant endocytosis. We note that while these are not the only parameters affecting α^*, they are the most important. Qualitatively α^* varies with these parameters in a manner similar to S

itself. This results because a high specificity allows for a large range of α over which to explore the dynamic range from $S = 1$ to the high maximum specificity, while a low specificity implies the opposite. This behavior appears to be robust across delivery protocols and endocytosis profiles.

References

1. Mulder A, Huskens J, Reinhoudt DN (2004) Multivalency in supramolucular chemistry and nanofabrication. Org Biomol Chem 2: 3409-3424.

2. Kolomeisky AB, Fisher ME (2000) Periodic sequential kinetic models with jumping, branching and deaths. Physica A 279: 1-20.
